# Efficacy and safety of omadacycline for the treatment of infectious diseases: a real-world, retrospective study of 2587 patients

**DOI:** 10.3389/fphar.2025.1706601

**Published:** 2025-11-18

**Authors:** Jian Liu, Jiayi Liu, Huangyi Li, Jiali Pang, Siyun Chen, Jianming Liu, Aijun Ouyang

**Affiliations:** 1 Department of Pharmacy, The First Affiliated Hospital, Jiangxi Medical College, Nanchang University, Nanchang, Jiangxi, China; 2 College of Pharmacy, Yichun University, Yichun, Jiangxi, China; 3 Department of Pharmacy, Affiliated Tumor Hospital of Guangxi Medical University, Nanning, Jiangxi, China; 4 Department of Pharmacy, Yongfeng County Traditional Chinese Medical Hospital, Ji’an, Jiangxi, China; 5 Department of Pharmacy, The People’s Hospital of Hezhou, Hezhou, Guangxi, China

**Keywords:** infectious diseases, omadacycline, efficacy, safety, predictors

## Abstract

**Objective:**

Previous randomized controlled trials have shown good efficacy and safety of omadacycline in patients with infectious diseases, but relevant real-world evidence is still insufficient. This large-scale study aimed to explore the efficacy and safety of omadacycline for the treatment of infectious diseases in real-world conditions.

**Methods:**

This was a retrospective, real-world study. A total of 2587 patients with infectious diseases who received omadacycline treatment were enrolled. Clinical success was defined as resolution or significant improvement of infection-related signs and symptoms without the need for salvage antimicrobial therapy.

**Results:**

After omadacycline treatment, 81.6% of patients achieved clinical success. Multivariate logistic regression analysis suggested that older age [odds ratio (OR): 0.991, *P* = 0.025], history of any malignancy (OR: 0.630, *P* = 0.016), abnormal renal function (OR: 0.432, *P* = 0.003), mechanical ventilation (OR: 0.559, *P* = 0.013), intensive care unit (ICU) (OR: 0.328, *P* < 0.001), and longer length of stay (LOS) (OR: 0.941, *P* < 0.001) were independently related to a lower probability of achieving clinical success. However, longer days of omadacycline use were independently associated with a higher probability of achieving clinical success (OR: 1.121, *P* < 0.001). Regarding adverse events, the incidence of coagulation disorders, acute kidney injury, liver injury, and gastrointestinal reaction was 10.5%, 9.9%, 6.2%, and 5.3%, respectively.

**Conclusion:**

Omadacycline possesses favorable efficacy and safety for the treatment of infectious diseases. Baseline age, malignancy history, renal function, mechanical ventilation, ICU, LOS, and omadacycline use duration are strong predictors of clinical success.

## 1 Introduction

Infectious diseases caused by bacteria, such as pneumonia, urinary tract infections, and skin and skin-structure infections, constitute a profound global health burden (Spagnolo, 2024). The primary pharmacological agents for treating bacterial infections include beta-lactams, macrolides, fluoroquinolones, and tetracyclines (Sujith et al., 2024; Vaughn et al., 2024; Tiseo and Falcone, 2025). However, some pathogens, such as *Enterococcus faecium*, *Acinetobacter baumannii*, *Klebsiella pneumoniae*, and *Staphylococcus aureus*, are becoming increasingly resistant to conventional antimicrobial agents, leading to poor clinical outcomes (Ho et al., 2025; Aggarwal et al., 2024; Krishnaprasad and Kumar, 2024; Larkin, 2023). It is estimated that bacterial antimicrobial resistance contributed to 1.14 million deaths globally in 2021, and projections suggest that this number will rise to 1.91 million in 2050 (GBDAR, 2024). Therefore, exploring promising antibiotics for the treatment of infectious diseases is fundamental.

Omadacycline, a novel aminomethylcycline belonging to the tetracycline class, is effective against a broad range of Gram-positive and select Gram-negative pathogens, including resistance determinant-containing strains (Macone et al., 2014; Karvouniaris et al., 2023; Kounatidis et al., 2024). Currently, several studies have demonstrated favorable efficacy and safety profiles of omadacycline in treating infectious diseases, including community-acquired bacterial pneumonia and acute bacterial skin and skin-structure infections (Torres et al., 2021; O'Riordan et al., 2019a; Stets et al., 2019; O'Riordan et al., 2019b; Gao et al., 2024). However, the majority of evidence is derived from randomized controlled trials (Torres et al., 2021; O'Riordan et al., 2019a; Stets et al., 2019; O'Riordan et al., 2019b), which employed stringent patient selection criteria. Such strict inclusion criteria limit the extrapolation of these findings to routine clinical settings. Although two real-world studies have been performed (Gao et al., 2024; El Ghali et al., 2023), the sample size was small, with one enrolling 183 patients (Gao et al., 2024) and the other enrolling 75 patients (El Ghali et al., 2023), which restricts the statistical power.

Accordingly, we established a large retrospective cohort and aimed to explore the efficacy and safety of omadacycline for the treatment of infectious diseases in real-world conditions.

## 2 Methods

### 2.1 Study design and population

This was a retrospective, real-world study that consecutively included 2587 adult patients who were treated with intravenous omadacycline (loading dose: 0.2 g once daily or 0.1 g twice daily; maintenance dose: 0.1 g once daily) between April 2022 and December 2024. To justify the representativeness of our study population in real world conditions, our cohort was drawn from diverse hospital departments, including respiratory and critical care medicine, public health medicine, emergency, infectious diseases, and general practice. Moreover, it encompassed a wide spectrum of infection sites, including pulmonary infections, urinary infections, skin and soft tissue infections, and others. Patients were included if they were: 1) age ≥18 years; 2) had a clinically and/or microbiologically confirmed diagnosis of infectious diseases, including but not limited to pulmonary infection, urinary infection, or skin and soft tissue infection; 3) received omadacycline as part of their antimicrobial therapy (either as monotherapy or in combination with other antimicrobials); 4) had complete baseline clinical data; 5) had available information for efficacy or safety assessment. Exclusion criteria were as follows: 1) died within 48 h of starting omadacycline treatment; 2) early discontinuation for other reason (e.g., financial or administrative issues) within 48 h; 3) received omadacycline therapy for less than 72 h. Although this was a single-center study, the hospital serves as a major tertiary referral center that manages a broad range of infectious diseases, and the included cases reflect the diversity and complexity of real-world patients receiving omadacycline. The Ethical Committee approved this study with the approval number of IIT20250347, and the waiver of informed consent was also approved. To protect patient privacy, all clinical data were de-identified before analysis: personal identifiers were permanently deleted, and only study-relevant clinical/treatment variables were retained. De-identified data were stored on the hospital’s encrypted server, with access limited to authorized research team members. All data management followed the Declaration of Helsinki and local medical data privacy regulations.

### 2.2 Data collection and variables

Clinical characteristics were screened from the Electronic Medical Record. The specific variables were as follows: general data (e.g., demographic, admission year, department of hospitalization), disease history (e.g., history of surgery, history of any malignancy), comorbid conditions (e.g., hypertension, coronary heart disease (CHD), diabetes mellitus (DM), septic shock, abnormal liver function, abnormal renal function, intracranial hemorrhage), and treatment information (e.g., infection site, treatment type, omadacycline form, days of omadacycline use, concomitant use of other antibiotics, mechanical ventilation, intensive care unit (ICU), and length of stay (LOS)). Laboratory data before and after treatment were extracted, involving liver function, renal function, and coagulation parameters.

### 2.3 Study outcomes

The efficacy outcome was clinical success, defined as resolution or significant improvement of infection-related signs and symptoms without the need for salvage antimicrobial therapy (Gao et al., 2024). Assessment of clinical success was conducted during the time window spanning from 72 h after the first dose to 7 days after the final administration of omadacycline. Besides, liver injury, acute kidney injury, gastrointestinal reaction, and coagulation disorders were screened for safety analysis (Technology Committee on DP et al., 2023; National Clinical Research Center for Kidney D et al., 2023; Author anonymous, 2024). All laboratory measurements in this study were performed using fully automated, clinically validated analyzers that were routinely used in our hospital’s clinical laboratory, including the Mindray BC-7500 for complete blood count, the Sysmex CN-6000 for coagulation tests, and the Roche cobas 8000 for biochemical parameters.

### 2.4 Statistics

The sample size was not calculated, and patients were consecutively included. Descriptive statistics were performed to describe the data. Continuous variables were summarized using medians with interquartile ranges (IQR). Between-group comparisons were conducted using the Mann-Whitney U test. The Wilcoxon signed-rank test was employed to compare paired data before and after omadacycline use, while unpaired data before and after omadacycline use were conducted via Mann-Whitney U test. Categorical variables were described as counts and corresponding percentages. Comparisons between categorical variables were assessed via the *Chi*-square test. Logistic regression analysis was conducted to explore the association between clinical characteristics and clinical success. Variables of general data, disease histories, comorbid conditions, and treatment information were included in a multivariate logistic regression model (forward step-wise method) to explore independent predictors of clinical success and adverse events. To minimize selection bias, we employed a consecutive enrollment strategy for all eligible patients. Measurement bias was minimized by using validated tools and pre-defined criteria for all assessments. Confounding was addressed by including the relevant covariates collected in multivariate regression analyses to adjust for potential baseline differences among patients. SPSS V.29.0 (IBM, United States of America) was used for data analysis. A *P* value <0.05 indicated statistical significance.

## 3 Results

### 3.1 Clinical features and treatment information

The median (IQR) age of patients was 66.0 (53.0–75.0) years. There were 1558 (60.2%) males and 1029 (39.8%) females. A total of 234 (9.0%) patients had a history of any malignancy. Regarding comorbidities, 781 (30.2%), 106 (4.1%), 342 (13.2%), 29 (1.1%), 266 (10.3%), 105 (4.1%), and 62 (2.4%) patients had hypertension, CHD, DM, septic shock, abnormal liver function, abnormal renal function, and intracranial hemorrhage, respectively. The detailed clinical features of patients are shown in Table 1.

**TABLE 1:** Clinical characteristics of patients receiving omadacycline treatment.

Items	Patients (N = 2587)
General data	
Age (years), median (IQR)	66.0 (53.0–75.0)
Sex, n (%)	
Male	1558 (60.2)
Female	1029 (39.8)
BMI (kg/m^2^), median (IQR) (n = 2123)	22.0 (19.6–24.7)
Admission year, n (%)	
2022	13 (0.5)
2023	986 (38.1)
2024	1588 (61.4)
Department of hospitalization, n (%)	
Respiratory and critical care medicine	875 (33.8)
Public health medicine	758 (29.3)
Emergency	266 (10.3)
Infectious diseases	204 (7.9)
General practice	85 (3.3)
Others	400 (15.5)
Disease histories	
History of surgery, n (%)	1024 (39.6)
History of any malignancy, n (%)	234 (9.0)
Comorbid conditions	
Hypertension, n (%)	781 (30.2)
CHD, n (%)	106 (4.1)
DM, n (%)	342 (13.2)
Septic shock, n (%)	29 (1.1)
Abnormal liver function, n (%)	266 (10.3)
Abnormal renal function, n (%)	105 (4.1)
Intracranial hemorrhage, n (%)	62 (2.4)

IQR, interquartile range; BMI, body mass index; CHD, coronary heart disease; DM, diabetes mellitus.

Regarding treatment-related information, the median (IQR) duration of omadacycline use was 8.0 (6.0–11.0) days. Mechanical ventilation was applied in 200 (7.7%) patients. Three hundred and sixty-three (14.0%) patients were admitted to the ICU. The median (IQR) LOS was 13.0 (9.0–19.0) days. The detailed treatment information is shown in Table 2. A total of 14.1% of patients used other antibiotics in combination, of which, β-lactam (35.3%) and carbapenems (27.4%) were the most common concomitant antibiotics. The detailed information on the concomitant antibiotics is shown in Supplementary Table S1.

**TABLE 2:** Treatment information of patients receiving omadacycline treatment.

Items	Patients (N = 2587)
Infection site, n (%)	
Pulmonary infection	2183 (84.4)
Urinary infection	74 (2.9)
Skin and soft tissue infection	52 (2.0)
Others or unknown infection	359 (13.9)
Treatment type, n (%)	
Targeted	461 (17.8)
Empiric	2126 (82.2)
Omadacycline form, n (%)	
Injection	2587 (100.0)
Days of omadacycline use (days), median (IQR)	8.0 (6.0–11.0)
Concomitant use of other antibiotics, n (%)	
No	2222 (85.9)
Yes	365 (14.1)
Mechanical ventilation, n (%)	
No	2387 (92.3)
Yes	200 (7.7)
ICU, n (%)	
No	2224 (86.0)
Yes	363 (14.0)
LOS (days), median (IQR)	13.0 (9.0–19.0)

IQR, interquartile range; ICU, intensive care unit; LOS, length of stay.

### 3.2 Pathogens

A total of 463 patients were microbiologically confirmed infections, and 580 pathogens were detected. The leading pathogens identified were *K. pneumoniae* (18.6%), followed by *Mycoplasma pneumoniae* (14.3%), *A. baumannii* (13.8%), *Pseudomonas aeruginosa* (6.9%) and *Stenotrophomonas maltophilia* (6.0%). The detailed pathogen profile is shown in Supplementary Table S2.

### 3.3 Clinical success rate after omadacycline treatment

**FIGURE 1:**
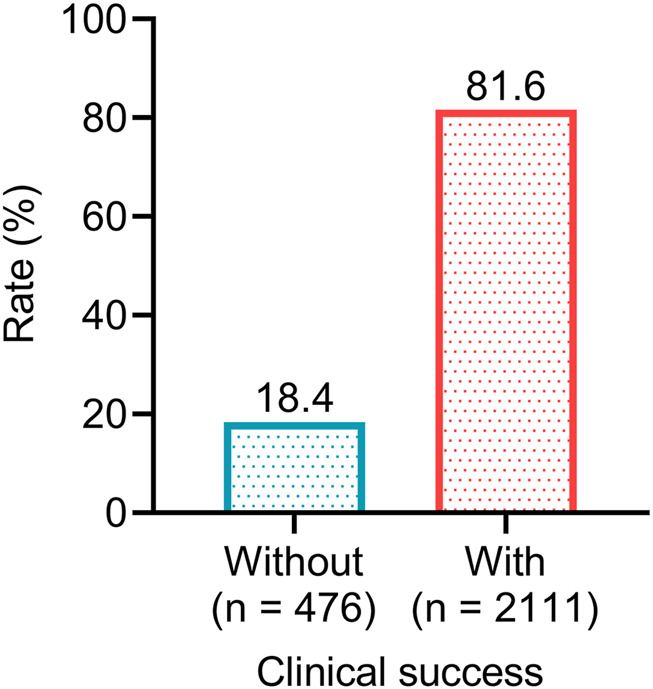
The proportions of patients achieving clinical success after omadacycline treatment.

After omadacycline treatment, a total of 2,111 patients achieved resolution or significant improvement of infection-related signs and symptoms, and did not require salvage antimicrobial therapy, resulting in a clinical success rate of 81.6%. The other 476 (18.4%) patients failed to achieve clinical success (Figure 1). The above data suggested that the efficacy of omadacycline was acceptable for the treatment of infectious diseases.

### 3.4 Factors related to clinical success after omadacycline treatment

**FIGURE 2:**
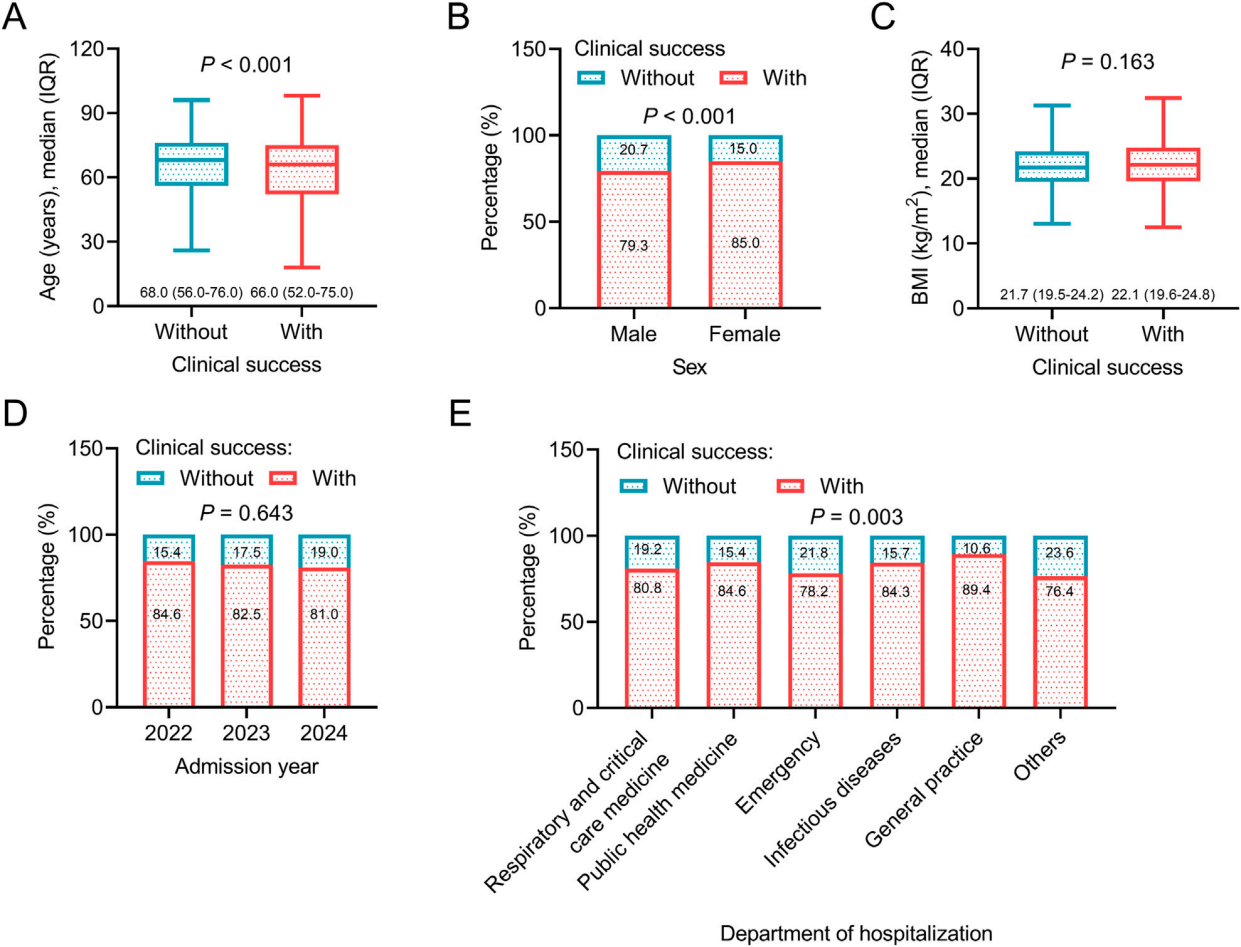
Association between general data and clinical success. Association of age **(A)**, sex **(B)**, BMI **(C)**, admission year **(D)**, and department of hospitalization **(E)** with clinical success in patients receiving omadacycline.

**FIGURE 3:**
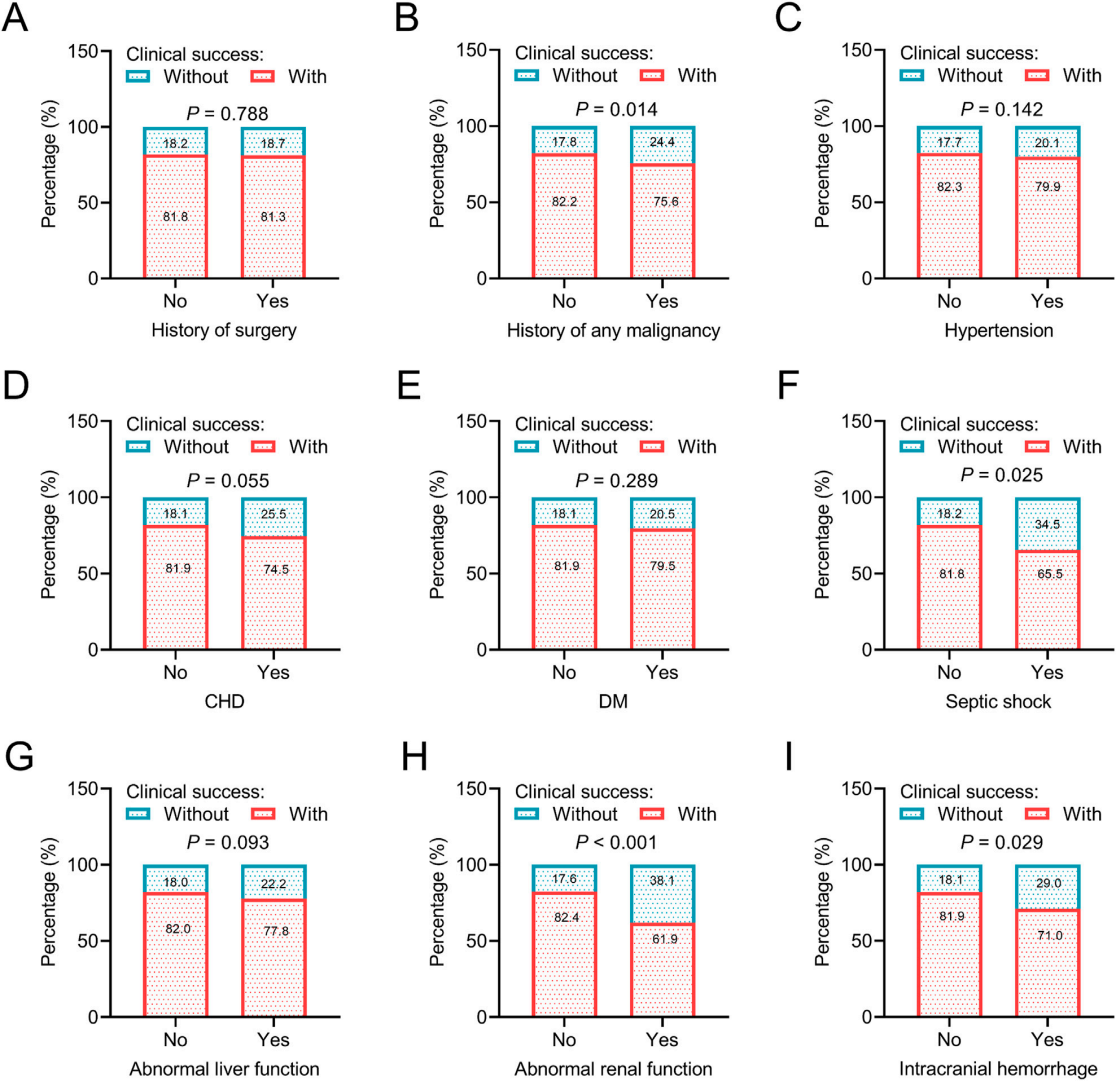
Association of disease histories and comorbid conditions with clinical success. Association of history of surgery **(A)**, history of any malignancy **(B)**, hypertension **(C)**, CHD **(D)**, DM **(E)**, septic shoch **(F)**, abnormal liver function **(G)**, abnormal renal function **(H)**, and intracranial hemorrhage **(I)** with clinical success in patients receiving omadacycline.

Regarding general data, younger age (*P* < 0.001), female sex (*P* < 0.001), and general practice department of hospitalization (*P* = 0.003) were associated with a higher rate of clinical success, which indicated that patients with the above characteristics might benefit more from omadacycline treatment. However, body mass index (BMI) and admission year were not associated with clinical success (both *P* > 0.05) (Figures 2A–E). Regarding disease histories and comorbid conditions, without a history of any malignancy (*P* = 0.014), without septic shock (*P* = 0.025), without abnormal renal function (*P* < 0.001), and without intracranial hemorrhage (*P* = 0.029) were related to a higher rate of clinical success. The above data suggested that patients without the major organ dysfunction and critical illness were more likely to achieve clinical success after omadacycline treatment. History of surgery and other comorbid conditions, including hypertension, CHD, DM, and abnormal liver function were not related to clinical success (all *P* > 0.05) (Figures 3A–I).

**FIGURE 4:**
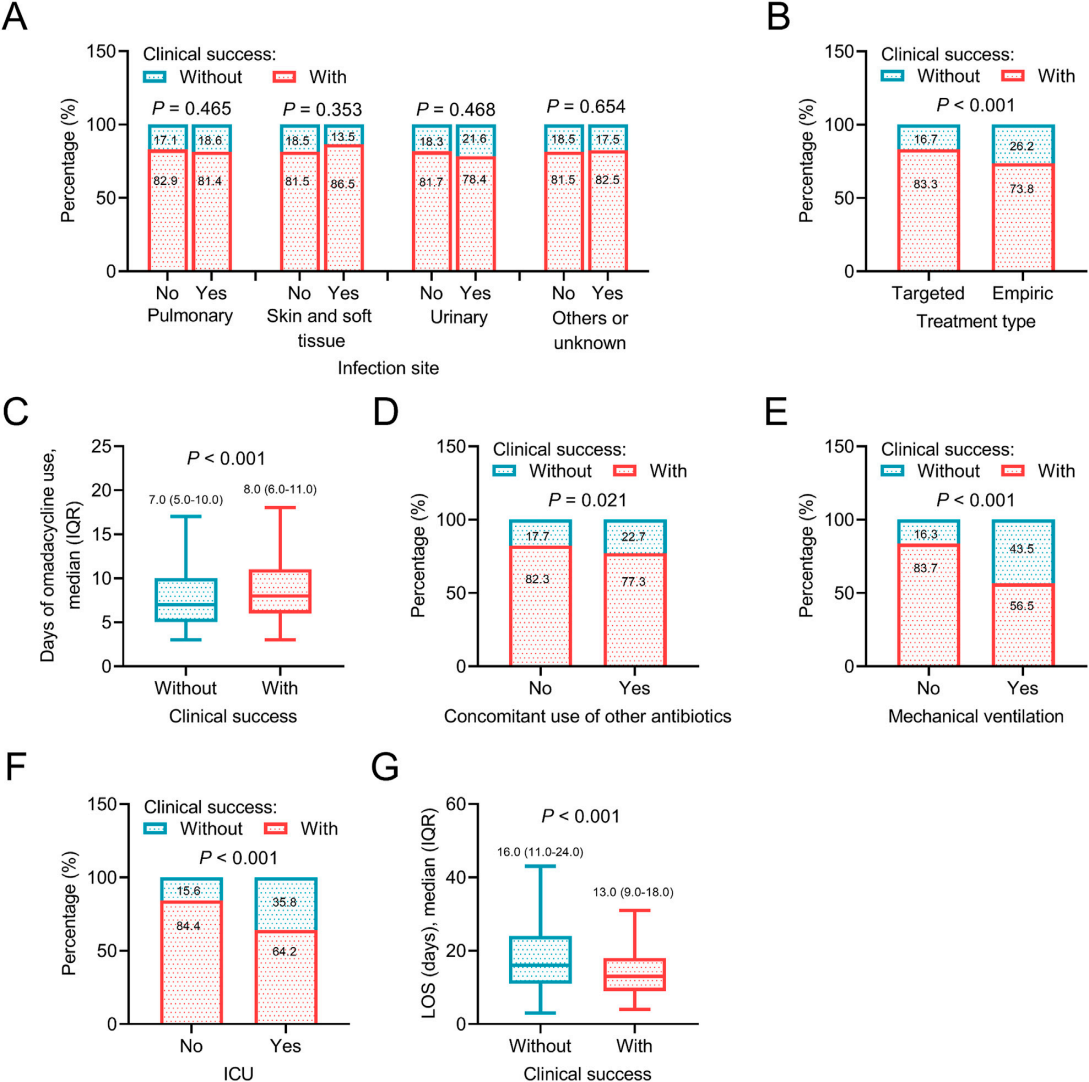
Association between treatment information and clinical success. Association of infection site **(A)**, treatment type **(B)**, days of omadacycline use **(C)**, concomitant use of other antibiotics **(D)**, mechanical ventilation **(E)**, ICU **(F)**, and LOS **(G)** with clinical success in patients receiving omadacycline.

In terms of treatment-related information, targeted treatment type (*P* < 0.001), longer days of omadacycline use (*P* < 0.001), without concomitant use of other antibiotics (*P* = 0.021), without mechanical ventilation (*P* < 0.001), not admitting in ICU (*P* < 0.001), and shorter LOS (*P* < 0.001) were related to a higher rate of clinical success, which suggested that adequate treatment course and less severe clinical status at baseline might be determinants for achieving clinical success after omadacycline treatment. However, the infection sites were not related to clinical success (all *P* > 0.05) (Figures 4A–G).

### 3.5 Multivariate logistic regression analysis for clinical success after omadacycline treatment

According to the multivariate logistic regression analysis, older age [odds ratio (OR): 0.991, *P* = 0.018], history of any malignancy (OR: 0.587, *P* = 0.004), abnormal renal function (OR: 0.423, *P* = 0.002), targeted treatment type (OR: 0.725, *P* = 0.035) mechanical ventilation (OR: 0.419, *P* < 0.001), and ICU (OR: 0.403, *P* < 0.001) were independently related to a lower probability of achieving clinical success. The above factors might serve as crucial predictors of clinical success after omadacycline treatment. Other factors, including concomitant use of other antibiotics, were not independently related to clinical success (Table 3).

**TABLE 3:** Multivariate logistic regression analysis on clinical success.

Factors	*P* value	OR	95% CI	
			Lower	Upper
Age (per year)	0.018	0.991	0.984	0.998
History of any malignancy (yes vs. no)	0.004	0.587	0.409	0.843
Abnormal renal function (yes vs. no)	0.002	0.423	0.243	0.738
Treatment type (targeted vs. empiric)	0.035	0.725	0.537	0.978
Mechanical ventilation (yes vs. no)	<0.001	0.419	0.270	0.650
ICU (yes vs. no)	<0.001	0.403	0.287	0.566

OR, odds ratio; CI, confidence interval; ICU, intensive care unit; LOS, length of stay. Identifier information, disease histories, comorbid conditions, and treatment information were input into the model. Days of omadacycline use and length of stay were not put in the model due to causal inversion problem. A forward step-wise multivariate logistic regression analysis was conducted.

### 3.6 The change in blood routine examination parameters after omadacycline treatment

In the unpaired analysis, after omadacycline treatment, improvements in blood routine examination parameters were noted (all *P* < 0.001). The paired analysis showed that after omadacycline treatment, white blood cell (WBC), hemoglobin, neutrophil, and C-reactive protein (CRP) were decreased, while platelet (PLT) and eosinophils were increased (all *P* < 0.001) (Table 4). The above findings indicated a significant resolution of inflammation following omadacycline treatment.

**TABLE 4:** Parameters of blood routine examination.

Items	Before medication		After medication		*P* value
	n (%)	Values	n (%)	Values	
Unpaired					
WBC (×10^9^/L), median (IQR)	2495 (96.4)	8.5 (5.9–11.7)	2537 (98.1)	6.7 (4.9–9.1)	<0.001
Hemoglobin (g/L), median (IQR)	2490 (96.3)	115.0 (98.0–130.0)	2544 (98.3)	110.0 (91.0–125.0)	<0.001
PLT (×10^9^/L), median (IQR)	2511 (97.1)	201.0 (135.0–271.0)	2543 (98.3)	253.0 (174.0–342.0)	<0.001
Neutrophil (%), median (IQR)	2497 (96.5)	80.9 (72.6–87.5)	2537 (98.1)	70.3 (59.4–80.8)	<0.001
Eosinophils (×10^9^/L), median (IQR)	2473 (95.6)	0.03 (0.00–0.11)	2527 (97.7)	0.11 (0.04–0.20)	<0.001
CRP (mg/L), median (IQR)	2465 (95.3)	66.9 (24.4–122.3)	2457 (95.0)	11.6 (4.3–40.8)	<0.001
Paired					
WBC (×10^9^/L), median (IQR)	2454 (94.9)	8.5 (5.9–11.7)	2454 (94.9)	6.7 (4.9–9.1)	<0.001
Hemoglobin (g/L), median (IQR)	2454 (94.9)	115.0 (98.0–130.0)	2454 (94.9)	110.0 (92.0–125.0)	<0.001
PLT (×10^9^/L), median (IQR)	2475 (95.7)	201.0 (135.0–271.0)	2475 (95.7)	253.0 (175.0–341.0)	<0.001
Neutrophil (%), median (IQR)	2456 (94.9)	81.0 (72.7–87.6)	2456 (94.9)	70.2 (59.2–80.8)	<0.001
Eosinophils (×10^9^/L), median (IQR)	2430 (93.9)	0.03 (0.00–0.11)	2430 (93.9)	0.11 (0.04–0.20)	<0.001
CRP (mg/L), median (IQR)	2355 (91.0)	68.0 (25.1–123.5)	2355 (91.0)	11.5 (4.3–40.1)	<0.001

WBC, white blood cell; IQR, interquartile range; PLT, platelet; CRP, C-reactive protein.

### 3.7 The change in liver function, kidney function, and coagulation function parameters after omadacycline treatment

In the unpaired analysis, most liver function, kidney function, and coagulation function parameters were numerically improved after omadacycline treatment (all *P* < 0.05), except for alkaline phosphatase (ALP) (*P* = 0.340) and D-dimer (*P* = 0.062). According to the paired analysis, liver function parameters, including aspartate aminotransferase (AST), total bilirubin (TBIL), and lactate dehydrogenase (LDH), were decreased, but alanine aminotransferase (ALT) was increased after omadacycline treatment (all *P* < 0.001). Regarding kidney function, creatinine (Cr), urea, and uric acid (UA) were decreased after omadacycline treatment (all *P* < 0.01). With respect to coagulation function, D-dimer, prothrombin time, and fibrinogen were reduced after omadacycline treatment (all *P* < 0.001) (Table 5). The above findings indicated that liver function, kidney function, and coagulation function were improved after omadacycline treatment.

**TABLE 5:** Parameters of liver function, kidney function, and coagulation function.

Items	Before medication		After medication		*P* value
	n (%)	Values	n (%)	Values	
Unpaired					
ALT (U/L), median (IQR)	1996 (77.2)	25.0 (14.2–49.6)	2413 (93.3)	31.0 (18.1–56.0)	<0.001
AST (U/L), median (IQR)	1999 (77.3)	30.0 (20.0–54.8)	2411 (93.2)	29.6 (20.3–44.8)	0.017
TBIL (μmol/L), median (IQR)	1991 (77.0)	10.0 (6.8–15.3)	2400 (92.8)	7.8 (5.4–11.6)	<0.001
GGT (U/L), median (IQR)	1872 (72.4)	34.3 (20.0–74.0)	2272 (87.8)	40.3 (23.0–81.6)	<0.001
ALP (U/L), median (IQR)	1896 (73.3)	85.5 (66.8–118.2)	2296 (88.8)	87.1 (66.8–119.9)	0.340
Cr (μmol/L), median (IQR)	1996 (77.2)	76.5 (60.1–103.0)	2383 (92.1)	69.9 (56.0–92.3)	<0.001
Urea (mmol/L), median (IQR)	1969 (76.1)	5.8 (4.1–9.0)	2338 (90.4)	5.3 (3.8–7.7)	<0.001
UA (μmol/L), median (IQR)	1972 (76.2)	252.0 (179.0–355.0)	2359 (91.2)	240.0 (171.3–322.0)	<0.001
LDH (U/L), median (IQR)	1605 (62.0)	275.7 (219.0–371.8)	1829 (70.7)	253.3 (202.4–333.7)	<0.001
D-dimer (mg/L), median (IQR)	1894 (73.2)	1.2 (0.5–3.0)	1784 (69.0)	1.1 (0.5–2.8)	0.062
PT (s), median (IQR)	1932 (74.7)	12.8 (11.9–13.9)	1846 (71.4)	12.3 (11.4–13.4)	<0.001
Fibrinogen (g/L), median (IQR)	1920 (74.2)	4.8 (3.5–6.1)	1821 (70.4)	3.8 (2.9–4.8)	<0.001
Paired					
ALT (U/L), median (IQR)	1888 (73.0)	25.5 (14.3–52.2)	1888 (73.0)	30.8 (18.0–56.0)	<0.001
AST (U/L), median (IQR)	1888 (73.0)	31.0 (20.0–56.2)	1888 (73.0)	30.6 (21.0–47.0)	<0.001
TBIL (μmol/L), median (IQR)	1875 (72.5)	10.1 (6.9–15.6)	1875 (72.5)	7.8 (5.4–11.9)	<0.001
GGT (U/L), median (IQR)	1711 (66.1)	35.0 (20.0–76.8)	1711 (66.1)	41.0 (23.1–83.0)	0.187
ALP (U/L), median (IQR)	1738 (67.2)	86.0 (67.0–119.0)	1738 (67.2)	88.4 (67.2–121.4)	0.404
Cr (μmol/L), median (IQR)	1869 (72.2)	76.8 (60.6–104.6)	1869 (72.2)	70.5 (55.8–95.2)	<0.001
Urea (mmol/L), median (IQR)	1826 (70.6)	5.9 (4.1–9.2)	1826 (70.6)	5.6 (3.9–8.2)	<0.001
UA (μmol/L), median (IQR)	1829 (70.7)	252.3 (180.0–356.5)	1829 (70.7)	233.9 (164.6–319.2)	<0.001
LDH (U/L), median (IQR)	1189 (46.0)	281.4 (222.6–391.0)	1189 (46.0)	257.4 (201.9–341.6)	<0.001
D-dimer (mg/L), median (IQR)	1411 (54.5)	1.5 (0.6–3.7)	1411 (54.5)	1.2 (0.5–3.0)	<0.001
PT (s), median (IQR)	1461 (56.5)	12.9 (12.0–14.1)	1461 (56.5)	12.4 (11.5–13.5)	<0.001
Fibrinogen (g/L), median (IQR)	1438 (55.6)	4.7 (3.5–6.0)	1438 (55.6)	3.8 (2.9–4.8)	<0.001

ALT, alanine aminotransferase; IQR, interquartile range; AST, aspartate aminotransferase; TBIL, total bilirubin; GGT, gamma-glutamyl transferase; ALP, alkaline phosphatase; Cr, creatinine; CKD-EPI, chronic kidney disease epidemiology collaboration; UA, uric acid; LDH, lactate dehydrogenase; PT, prothrombin time.

### 3.8 Safety

The most common adverse event was coagulation disorders (10.5%), followed by acute kidney injury (9.9%), liver injury (6.2%), and gastrointestinal reaction (5.3%). Most liver injury was grade I-II (5.9%), with only 0.3% being grade III. For acute kidney injury, the incidence was 5.5% for grade I-II and 4.4% for grade III (Table 6). These safety data suggested that omadacycline possessed a tolerable safety profile in real-world practice.

**TABLE 6:** Treatment-related adverse events.

Items	Total	Grade I	Grade II	Grade III
Drug-induced liver injury, n (%)	160 (6.2)	127 (4.9)	25 (1.0)	8 (0.3)
Acute kidney injury, n (%)	62 (2.4)	27 (1.0)	18 (0.7)	17 (0.7)
Coagulation disorders, n (%)	271 (10.5)	(-)	(-)	(-)

### 3.9 Multivariate logistic regression analysis for adverse events after omadacycline treatment

Older age (OR: 1.020, *P* < 0.001), abnormal liver function (OR: 2.030, *P* < 0.001), abnormal renal function (OR: 6.628, *P* < 0.001), targeted treatment type (OR: 1.374, *P* = 0.013), mechanical ventilation (OR: 2.265, *P* < 0.001), and ICU (OR: 3.458, *P* < 0.001) were independently related to a higher risk of adverse events. The above factors might serve as crucial predictors of adverse events after omadacycline treatment. Other factors, including concomitant use of other antibiotics, were not independently related to adverse events (Table 7).

**TABLE 7:** Multivariate logistic regression analysis on adverse events.

Factors	*P* value	OR	95% CI	
			Lower	Upper
Age (per year)	<0.001	1.020	1.014	1.026
Abnormal liver function (yes vs. no)	<0.001	2.030	1.468	2.806
Abnormal renal function (yes vs. no)	<0.001	6.628	3.198	13.737
Treatment type (targeted vs. empiric)	0.013	1.374	1.069	1.766
Mechanical ventilation (yes vs. no)	<0.001	2.265	1.416	3.623
ICU (yes vs. no)	<0.001	3.458	2.463	4.854

OR, odds ratio; CI, confidence interval; ICU, intensive care unit. Identifier information, disease histories, comorbid conditions, and treatment information (excepted for ‘days of omadacycline use’ and ‘length of stay’) were input into the model. A forward step-wise multivariate logistic regression analysis was conducted.

## 4 Discussion

Previous *in vitro* experiments have disclosed that omadacycline is active against various bacteria and antibiotic-resistant bacteria (Nicklas et al., 2022; Heine et al., 2024; Waites et al., 2022; Li et al., 2023; Skinner et al., 2023). Clinically, omadacycline demonstrates favorable efficacy for the treatment of diverse infectious diseases (Torres et al., 2021; O'Riordan et al., 2019a; Stets et al., 2019; O'Riordan et al., 2019b; Gao et al., 2024). For instance, two randomized controlled trials reported that omadacycline achieved a clinical success rate of 87.6%–88.4% in patients with community-acquired bacterial pneumonia (Torres et al., 2021; Stets et al., 2019). The other two previous randomized controlled trials indicated that the clinical success rate ranged from 84.0% to 86.1% after omadacycline treatment in patients with acute bacterial skin and skin-structure infections (O'Riordan et al., 2019a; O'Riordan et al., 2019b). Regarding patients with urinary tract infection, a phase 1b study found that the clinical success rate on 5–9 days after the last omadacycline dosing was 84.0% (Overcash et al., 2019). In this study, we found that after omadacycline treatment, 81.6% of patients with infectious diseases achieved clinical success. Our findings were in line with the previous trials, which suggested that omadacycline could be considered as a potent antibiotic for treating patients with infectious diseases. In real-world conditions, a previous study found that the clinical success rate was 71.0% after omadacycline treatment in patients with infectious diseases (Gao et al., 2024). Another study reported that 80.0% of patients with infectious diseases achieved clinical success after omadacycline treatment (El Ghali et al., 2023). The clinical success rate after omadacycline treatment seemed slightly different between our study and previous real-world studies. We speculated that the disparities might be attributed to variations in patients’ baseline clinical characteristics and treatment information. Overall, these collective data support that omadacycline had a good ability to relieve infection-related signs and symptoms, even under complex real-world clinical conditions.

According to a previous study, several factors were related to clinical success after omadacycline treatment in patients with infectious diseases, including moderate to severe liver function impairment, admission to the respiratory department, and omadacycline treatment duration (Gao et al., 2024). In this study, we performed a multivariate logistic regression analysis and discovered several independent factors related to clinical success after omadacycline treatment. (Spagnolo, 2024). Older age was independently related to a lower probability of clinical success. This finding suggested that younger patients might benefit more from omadacycline treatment, which may be related to their better immune function (Liu et al., 2023). (Sujith et al., 2024) History of any malignancy was independently related to a lower probability of clinical success. A potential reason might be that malignancies and their treatments might impair patients’ immune function, weakening the ability to fight infections (Lehrnbecher et al., 2008), thereby reducing the likelihood of clinical success after omadacycline treatment. (Vaughn et al., 2024). Abnormal renal function was independently related to a lower probability of clinical success after omadacycline treatment. A possible explanation would be that renal dysfunction often associates with immune impairment (Bumbea et al., 2024), which might weaken the body’s ability to clear bacteria, thereby preventing clinical success. (Tiseo and Falcone, 2025). Mechanical ventilation and ICU were independently associated with a lower probability of clinical success. This finding revealed that patients with severe disease conditions were more prone to treatment failure. Therefore, individualized treatment strategies and close monitoring are warranted for these patients.

Omadacycline possesses a good safety profile for the treatment of infectious diseases according to previous studies (Torres et al., 2021; O'Riordan et al., 2019a; Stets et al., 2019; O'Riordan et al., 2019b; Mingora et al., 2023). In this study, we found that inflammation-related parameters were improved after omadacycline treatment. This finding was supported by previous studies, which indicated that omadacycline possessed anti-inflammatory activities (Gomi et al., 2025; Sanders and Beringer, 2024). Apart from inflammation, we also found that most of the liver, kidney, and coagulation function-related parameters were improved after omadacycline treatment. However, we noted that ALT was elevated after omadacycline treatment in patients with infectious diseases. According to previous studies, increased ALT was a common phenomenon after omadacycline treatment (Torres et al., 2021; O'Riordan et al., 2019a; Stets et al., 2019; O'Riordan et al., 2019b). Additionally, the median ALT after omadacycline treatment was still within the normal range. Thus, this slight ALT elevation might not indicate a potential hepatotoxicity risk of omadacycline. It should be clarified that while our study observed statistically significant changes in multiple laboratory parameters after omadacycline treatment, the clinical relevance of many of these changes was limited. Previous studies reported that gastrointestinal reaction was a common adverse event after omadacycline treatment, with an incidence of 10.2%–28.0% in patients with infectious diseases (Stets et al., 2019; O'Riordan et al., 2019b; El Ghali et al., 2023). In this study, we found that the incidence of gastrointestinal reaction was only 5.3% in patients with infectious diseases receiving omadacycline. Other adverse events included coagulation disorders, acute kidney injury, and liver injury. Notably, coagulation disorders were relatively common after omadacycline treatment, with an incidence of 10.5%. A potential reason might be that tetracycline could suppress the activity of plasma prothrombin, which might further contribute to the occurrence of coagulation disorders (Cui et al., 2019). However, most cases of coagulation disorders in this study were mild (asymptomatic) based on our clinical observations, and no patients required special treatments. Regarding liver injury and acute kidney injury, most cases were mild to moderate. Overall, our study provided real-world evidence that omadacycline was safe and tolerable for the treatment of infectious diseases.

## 5 Limitations

Several limitations existed in this study. (Spagnolo, 2024). Selection bias and information bias might exist in this retrospective study, both of which could impair the representativeness of the patient population. Additionally, the completeness and accuracy of data could also be constrained by the quality of medical records. (Sujith et al., 2024). This study only included patients receiving omadacycline treatment and did not set a control group, such as patients treated with other antibiotics, which prevented us from comparing the efficacy and safety of omadacycline with other alternative antimicrobial agents. Therefore, the comparative advantages of omadacycline in treating infectious diseases should be further explored by subsequent studies. (Vaughn et al., 2024). This study focused on Chinese patients with infectious diseases. Therefore, the generalizability of our findings to patients from other regions was restricted. (Tiseo and Falcone, 2025). The definition of clinical success depended on the subjective judgement of clinicians, which might introduce evaluation bias. (Ho et al., 2025). This study lacked minimum inhibitory concentration (MIC) data for omadacycline against the isolated pathogens. As a real-world retrospective study, microbiological susceptibility testing for omadacycline was not routinely performed for all patients, which limited our ability to correlate clinical outcomes with *in vitro* susceptibility profiles. (Aggarwal et al., 2024). This study provided the incidence of adverse events after omadacycline treatment. However, it was difficult to distinguish whether these adverse events were caused by omadacycline due to retrospective design. Therefore, the incidence of treatment-related adverse events should be further explored by further prospective studies. (Krishnaprasad and Kumar, 2024). This study lacked resistance profiles for all pathogens because a substantial number of pathogens were detected by nucleic acid tests (without culture), which precluded conventional antimicrobial susceptibility testing. Lacking this data prevented us from exploring the efficacy of omadacycline in patients with infections caused by pathogens with a high level of resistance. (Larkin, 2023). The prespecified outcome of pathogen eradication rate was not reported in the final analysis. This was because a follow-up microbiological test to confirm pathogen clearance was not routinely performed for a substantial portion of the patients. The lack of this systematic follow-up data made it statistically unreliable to calculate the eradication rate.

## 6 Conclusion

In conclusion, our findings from a real-world cohort of 2,587 Chinese patients suggest that omadacycline possesses favorable efficacy and safety for the treatment of infectious diseases. Further studies incorporating control groups and diverse geographic populations are required to support the wide clinical application of omadacycline in patients with infectious diseases.

## Data Availability

The original contributions presented in the study are included in the article/Supplementary Material, further inquiries can be directed to the corresponding authors.
