# Correction: Efficacy and safety of omadacycline for the treatment of infectious diseases: a real-world, retrospective study of 2587 patients

**DOI:** 10.3389/fphar.2026.1842491

**Published:** 2026-05-04

**Authors:** Jian Liu, Jiayi Liu, Huangyi Li, Jiali Pang, Siyun Chen, Jianming Liu, Aijun Ouyang

**Affiliations:** 1 Department of Pharmacy, The First Affiliated Hospital, Jiangxi Medical College, Nanchang University, Nanchang, Jiangxi, China; 2 College of Pharmacy, Yichun University, Yichun, Jiangxi, China; 3 Department of Pharmacy, Affiliated Tumor Hospital of Guangxi Medical University, Nanning, Jiangxi, China; 4 Department of Pharmacy, Yongfeng County Traditional Chinese Medical Hospital, Ji’an, Jiangxi, China; 5 Department of Pharmacy, The People’s Hospital of Hezhou, Hezhou, Guangxi, China

**Keywords:** infectious diseases, omadacycline, efficacy, safety, predictors

There was a mistake in table Table 6 as published. We identified an error in Table 6 regarding the data on acute kidney injury (AKI). The verified and corrected data are as follows: total incidence 2.4%, grade I incidence 1.0%, grade II incidence 0.7%, and grade III incidence 0.7%.

The corrected table Table 6 appears below.

**TABLE 6:** Treatment-related adverse events.

Items	Total	Grade I	Grade II	Grade III
Drug-induced liver injury, n (%)	160 (6.2)	127 (4.9)	25 (1.0)	8 (0.3)
Acute kidney injury, n (%)	62 (2.4)	27 (1.0)	18 (0.7)	17 (0.7)
Coagulation disorders, n (%)	271 (10.5)	(-)	(-)	(-)

The original article has been updated.

