# Supplementary material for: Efficacy and safety of omadacycline for the treatment of infectious diseases: a real-world, retrospective study of 2587 patients
Source: Front Pharmacol. 2025 Nov 18;16:1706601. doi: 10.3389/fphar.2025.1706601 (PMC12669158; doi:10.3389/fphar.2025.1706601)
Supplement: Supplementary file 1 [file Table1.docx]

***Supplementary Materials***

**Supplementary Table 1.** Type of concomitant use of other antibiotics.

| Types | Patients (n = 365) |
| --- | --- |
| β-lactam | 129 (35.3) |
| Carbapenems | 100 (27.4) |
| Cephalosporins | 36 (9.9) |
| Antifungal agents | 29 (7.9) |
| Fluoroquinolones | 17 (4.7) |
| Oxazolidinones | 14 (3.8) |
| Glycopeptides | 14 (3.8) |
| Aminoglycosides | 6 (1.6) |
| Polymyxins | 5 (1.4) |
| Penicillins | 4 (1.1) |
| Sulfonamides | 3 (0.8) |
| Antiviral agents | 2 (0.5) |
| Glycylcyclines | 2 (0.5) |
| Tetracyclines | 2 (0.5) |
| Antituberculosis drugs | 1 (0.3) |
| Lipopeptides | 1 (0.3) |

**Supplementary Table 2.** Pathogen profile.

| Items | Patients (N=463) |
| --- | --- |
| **Pathogens, n (%)** |  |
| *Klebsiella pneumoniae* | 108 (18.6) |
| *Mycoplasma pneumoniae* | 83 (14.3) |
| *Acinetobacter baumannii* | 80 (13.8) |
| *Pseudomonas aeruginosa* | 40 (6.9) |
| *Stenotrophomonas maltophilia* | 35 (6.0) |
| *Escherichia coli* | 19 (3.3) |
| *Staphylococcus aureus* | 18 (3.1) |
| *Haemophilus influenzae* | 18 (3.1) |
| *Staphylococcus epidermidis* | 11 (1.9) |
| Others | 168 (29.0) |
